# Layering perspectives: a structured approach to meaningful patient and public involvement and engagement in the RETURN dental trial

**DOI:** 10.1186/s40900-026-00857-w

**Published:** 2026-03-07

**Authors:** V. Lowers, M. Stanley, J. Hennessy, E. Morgan, R. Horsley, J. Vithlani, R. V. Harris

**Affiliations:** 1 Department of Public Health, Policy and Systems, University of Liverpool, Whelan Building, 2nd Floor, Room 2.15, Brownlow Street, Liverpool, L69 3GQ UK; 2 Public Representative, Liverpool, UK

**Keywords:** Patient and public involvement, Co-production, Health inequalities, Dental research

## Abstract

**Background:**

Patient and public involvement and engagement (PPIE) is central to research addressing health inequalities and requires involvement that reflects the lived realities of those most affected. However, PPIE often relies on a narrow group of contributors, limiting socio-economic representation and raising concerns about its relevance for inequalities-focused research. To address this, a layered PPIE strategy was developed to increase the breadth and depth of involvement, and this paper presents a case study demonstrating its use in the RETURN dental study.

**Methods:**

A layered PPIE strategy was embedded within the RETURN study, comprising a lay researcher, a patient reference group of experienced PPIE contributors, a community advisory group representative of RETURN participants, and grassroots engagement activities designed to increase breadth and inclusivity by reaching individuals less likely to engage through formal PPIE routes. Each layer involved different types of activity and temporal arrangements, ranging from one-off engagement to sustained, longitudinal relationships. In total, over 300 PPIE collaborators contributed to the development of the RETURN study, including co-production of the intervention and patient-facing materials, refinement of patient information sheets and questionnaires, input into recruitment practices, and dissemination activities.

**Results:**

Each PPIE layer contributed distinct forms of input, with differing contributions, challenges, and lessons learned. Quotations and concrete examples from the RETURN study illustrate the impact of involvement across layers. Differences in perspectives across layers provided valuable insights and, taken together, enabled a more meaningful and inclusive form of PPIE than would have been achievable through a single layer alone.

**Conclusions:**

The layered PPIE strategy presented in this paper can strengthen inclusivity within health inequalities research by combining multiple forms of involvement aligned to different purposes and stages of the research. Engaging both experienced PPIE contributors and people less likely to be involved through conventional routes enabled perspectives from across the socio-economic spectrum, while preserving depth and rigour. Layered approaches offer a practical way to move beyond tokenism and support meaningful, context-sensitive involvement in complex research programmes.

**Trial registration:**

ISRCTN (http://www.isrctn.org/) 84666712, 12.01.2021

**Supplementary Information:**

The online version contains supplementary material available at 10.1186/s40900-026-00857-w.

## Abstract (de)

The RETURN trial explored how to help people who only go to the dentist when they are in pain. Regular visits are important for healthy teeth, but many people face dental anxiety, and those on lower incomes can face barriers like cost and time. To make sure our approach worked for real people, we involved the public at every stage. We used four layers of involvement:

- A **Lay Researcher** worked with the team to lead engagement and connect with local communities.
- A **Patient Reference Group** gave feedback on trial materials and processes.
- A **Community Advisory Group**, made up of people who had used urgent dental care, helped design the intervention and even appeared in videos and booklets.
- **Grassroots engagement** reached those who don’t usually take part in research, such as construction workers and young adults, through informal chats and creative activities in community spaces.

The intervention itself was based on a short conversation with a dental nurse, supported by videos and booklets that tackled common barriers like anxiety, cost, and lack of time. People were encouraged to set a goal for their next dental visit and received a follow-up text message as a reminder. This layered approach meant we heard from a wide range of voices, making the intervention practical and relevant. It wasn’t always easy building trust and adapting to different settings took time, however it showed that involving people meaningfully is possible when you listen, stay flexible, and take the time to conduct PPIE within existing community settings.

## Introduction

Regular dental attendance for check-ups and routine care is associated with reduced oral disease and better oral-health related quality of life [1, 2]. However, some people only visit a dentist when they have problems, and this is more likely among people from more disadvantaged backgrounds [3–5].

Although barriers to dental attendance are well documented [6], evidence on interventions to reduce attendance inequalities in adults remains limited [7]. Given this gap, interventions should be shaped by the people they aim to target to improve relevance, feasibility, and acceptability [8].

RETURN is a new intervention delivered to adults attending for unplanned urgent dental care that aims to reduce inequalities in planned dental attendance [9]. This paper outlines how a layered approach to patient public involvement and engagement (PPIE) shaped RETURN by widening socio-economic inclusion in its co-production.

## Background

PPIE is supported by a large and sometimes confusing landscape of overlapping models and guidance. Over 500 PPIE frameworks have been identified in the literature [10], which can encourage emphasis on definitions and terminology over the core purpose of involving people whose lives are affected by research [11]. While calls for “**nothing about us without us**” [12] have driven more democratic and inclusive approaches [13], PPIE is increasingly mandated by funders and journals [14, 15], raising concerns about tokenism [16–18].

Arnstein’s “Ladder of Citizen Participation” [19] conceptualises involvement as a progression towards increasing citizen power within civic decision-making. It has been widely applied to PPIE as a way of reflecting on power-sharing and decision making structures within health research contexts. Despite widespread recognition of the moral imperative [13, 20] and added value of meaningful PPIE in health research [21, 22], a gap remains between the ideal of co-production, positioned at the top of Arnstein’s ladder [19], and the realities of implementing this ideal within complex, often rigid research settings [11, 23].

Achieving meaningful involvement in inequalities-focused research requires the active inclusion of people from populations most affected [24]. However, evidence consistently shows that PPIE relies on contributors who could be considered the “usual suspects”: individuals who are well-educated, typically older, and from non-minority ethnic groups [25–27]. Individuals from under-represented groups are less likely to participate in research, and when they do, they may feel less confident that their contributions will be treated with dignity and respect [28]. This raises an important question for inequalities-focused PPIE: *can research involvement be truly meaningful if it fails to include voices from across the socio-economic spectrum*?

Barriers to PPIE inclusivity may be structural, cultural, procedural, or practical [23, 29]. Common pitfalls to meaningful PPIE include over-reliance on online methods (despite one third of the world’s population lacking internet access) [30] or recruitment strategies dependent on researchers’ personal or professional networks, which may introduce bias and reinforce exclusion [31]. Addressing these barriers requires context-sensitive, flexible approaches [22] that respond to differences between groups, such as taking activities into community settings rather than expecting contributors to attend formal meetings in academic environments [32].

Given the typical profile of PPIE collaborators in research, a further challenge in implementing inequalities-focused PPIE is that those ‘representing’ the lay voice often come from higher socio-economic backgrounds. Additionally, over time, perspectives may become increasingly shaped by involvement in research, creating a risk that some of the authenticity of the patient or public perspective is lost [33].

To address these challenges, the RETURN research programme deliberately incorporated multiple layers of PPIE, each with different aims and membership. Combining experienced contributors with those more “naïve” to research aimed to broaden representation while preserving authenticity of lived experience, in line with project objectives. This approach aligns with the UK Standards for Public Involvement, which emphasise flexible, proportionate PPIE, shaped by research needs [34].

This paper aims to describe (1) a layered PPIE approach and (2) challenges and lessons learned in navigating this multi-perspective approach. Our PPIE strategy is presented in accordance with the GRIPP2 (short form) reporting guidelines [35].

## Methods

### The RETURN project

RETURN was a staged research programme comprising ethnography to identify barriers to routine dental attendance [6], refinement of a prototype intervention [11] and testing and further refinement in a feasibility study [36]. The final intervention was evaluated in a randomised controlled trial and cost-effectiveness study, recruiting over 1100 people. RETURN was delivered by trained dental nurses in urgent dental services. It took around 15-minutes to deliver and involved motivational interviewing-informed conversations, short videos, and a set of booklets addressing barriers to dental attendance. Dental nurses also supported participants to set a dental attendance goal, reinforced with a text message [37].

### RETURN multi-layered patient and public involvement and engagement structure

The RETURN intervention targeted people attending urgent dental services, a group that includes a high proportion of individuals from socio-economically disadvantaged backgrounds. A single PPIE model was insufficient to capture the diversity of experiences represented within this population, and reliance on one group risked privileging some perspectives over others. Our strategy, therefore, sought to increase both the breadth and depth of PPIE to support co-design of RETURN to reduce inequalities in routine dental attendance.

We adopted a multi-layered PPIE approach, developed iteratively and shaped by insights from PPIE activity and other RETURN workstreams. Each layer had a distinct purpose, with insights generated in one layer informing discussion in the others.

#### Lay researcher(LR)

A lay person (MS) was employed for 2–3 days a week alongside RETURN researchers from 2018 to 2025. She was recruited as someone with no previous experience of research but who had strong communication skills. The objective of the LR role was to embed a sustained lay perspective and to coordinate and iteratively develop PPIE activity. The role included connecting with local people, articulating a lay perspective in research team discussions, coordinating PPIE layers, recording activities for reporting, and leading PPIE activity.

#### Patient reference group(PRG)

Several experienced lay representatives were recruited to provide feedback on study processes, materials, and dissemination. The objective of the PRG was to provide informed critique, drawing on members’ experiences of representing lay perspectives in research and community settings. PRG membership necessitated a level of literacy and capacity to navigate research processes. Their views helped inform the research and LR-led PPIE activities.

#### Community advisory group(CAG)

Since proportionally more urgent dental care attenders are from low socio-economic groups, we recruited a second PPIE group who were more representative of the intervention end-users. The objective of the CAG was to co-produce the intervention and patient-facing materials, ensuring that these were meaningful to those most likely to receive the intervention. The views and materials developed by the CAG informed PRG discussions, LR-led activities with grassroots groups (layer 4), and LR advice to guide research conduct decisions.

#### Grassroots engagement (GE) with community groups and the public

GE drew on pre-existing community and voluntary sector groups with longstanding and established relationships with disadvantaged communities in the local area. The objective of GE was to engage individuals who were representative of the intervention end-users, and to ensure that we reached individuals who would not have felt comfortable participating in more formal PPIE structures. The LR spent time engaging with community leaders and members in their own settings. Feedback from these interactions was discussed with the research team, the CAG and PRG.

**Fig. 1:**
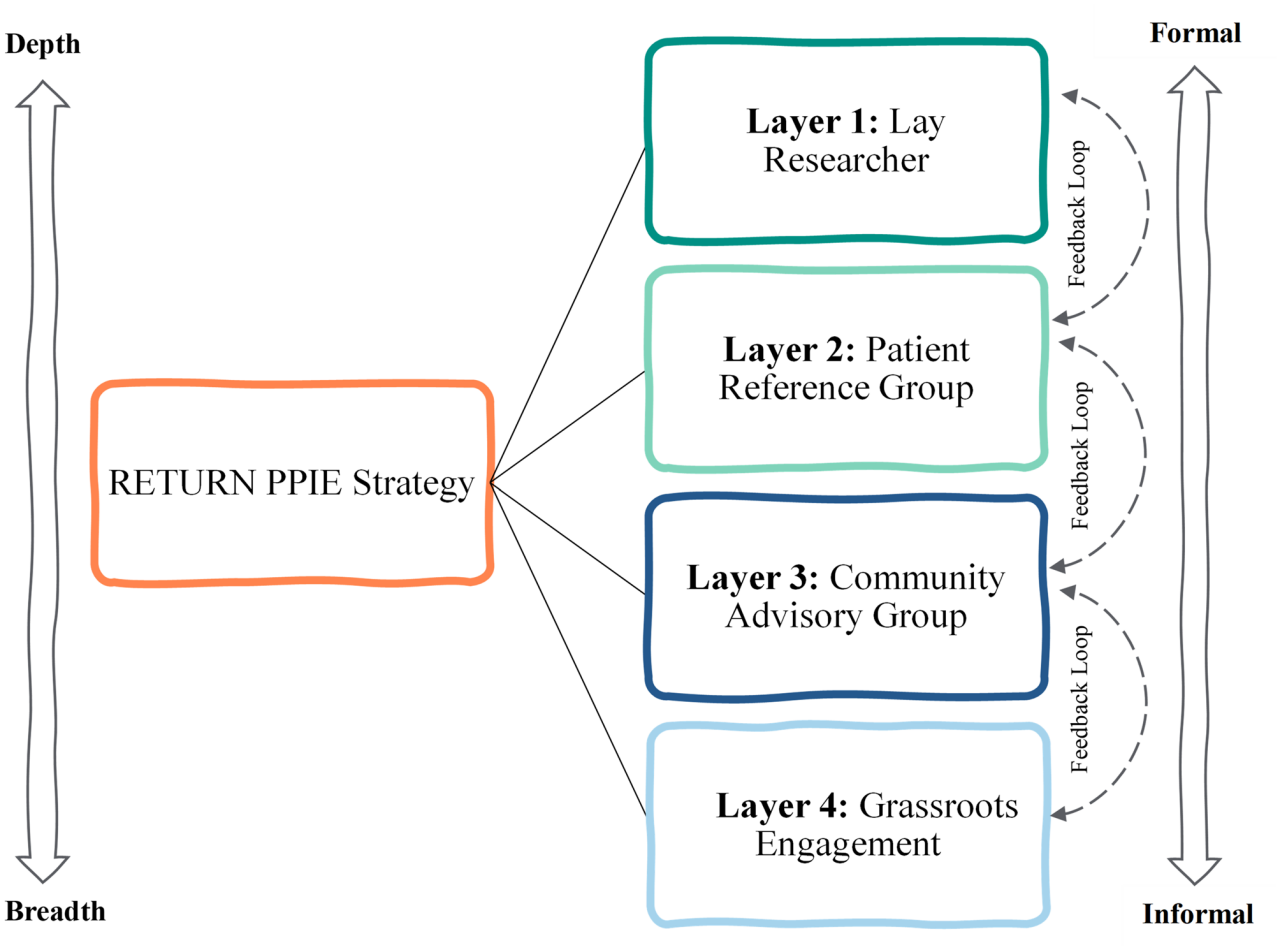
Layered PPIE strategy used in RETURN

The PPIE strategy is summarised in Fig. 1.

### Ethics and governance

In the UK, ethical approval is not mandatory for PPIE activities. However, ethical issues in this field are widely recognised, particularly when using creative engagement methods, and ethical guidance has been identified as a strategic priority [38]. Our strategy included participatory activities (e.g. photographs, journey mapping) and social media engagement. The grassroots approach meant involvement was sometimes unpredictable and could include vulnerable individuals, reinforcing the need for ethical oversight to ensure rigour, integrity, and transparency. We therefore obtained approval from the Central University of Liverpool Ethics Committee for Psychological Interventions (ref: 4021) to ensure transparency and best practice.

The LR completed training in ethics, confidentiality and participatory approaches to support contributors safely and respectfully. Where PPIE activities involved participatory approaches, the LR was supported by the research team. Opportunities to attend formal PPIE training were also offered. Written informed consent was obtained for ongoing PPIE activities, and verbal consent for one-off interactions. No personal data were collected during one-off interactions. Interactions were anonymously documented using audio transcripts, field notes, reflections, photographs, drawings, written feedback, or emails. Contributors were reimbursed in line with national guidance [39].

### Reflexivity and positionality

The LR role was positioned to provide an independent voice and ‘research naïve’ perspective, intended to counterbalance the positionality of the research team. Given the length of the programme (2018–2025), some degree of professional development was inevitable. During this period the LR completed a Master’s degree and became increasingly integrated within the research team. In recognition of this evolving professional identity, the LR’s job title was updated to PPIE Coordinator (although we have retained LR label here for consistency).

The wider RETURN research team comprised academic researchers and dental professionals based within a university setting. This institutional context, and the associated power differentials between researchers and contributors was recognised throughout. This informed decisions about how PPIE activities were designed and facilitated.

## Results

### Layer 1: the lay researcher

The LR was female, White British, in her 40s, from the local area and had a background in teaching. Although she did not mirror the socio-economic background of intervention users, she demonstrated insight into the factors influencing dental attendance and was able to build rapport in multiple contexts.

Leading PPIE activities involved more than simply “chatting to people.” As the LR reflected: *“It wasn’t just turning up and talking, it was about finding the right doors to knock on and sometimes pushing them open.”* She actively researched and identified who to approach, drawing on local urgent dental care utilisation data, wider literature, and direct feedback from PPIE contributors. These insights shaped an iterative, flexible outreach strategy aligned with project objectives. For example, although older, retired people were more likely to express interest in PPIE involvement, RETURN ethnographic work showed urgent care users were often younger (18–24-years) with many in zero-hours type employment. To engage individuals who more closely represented intervention end users, the LR refined the PPIE approach, prioritising groups identified through the ethnographic work.

We found two qualities were central to the success of this role:

- **Adaptability:** The LR recognised that a “one size fits all” approach would not succeed. She tailored recruitment strategies, activity types, and communication styles to each context. For groups with low literacy, she used visual and hands-on activities, including photographs, journey mapping, quizzes, and design workshops. In settings where distrust was a barrier, she first participated in group activities, such as playing football, before introducing project-related discussions. She worked iteratively and always consulted gatekeepers to ensure approaches were appropriate. She reflected: *“You have to meet people where they are—sometimes that means on the football pitch, not in a meeting room.”*
- **Tenacity:** Engaging individuals who are seldom represented in research co-design required persistence and creativity. In some cases, this meant physically attending unexpected locations that were not traditional research spaces. For example, visiting a building site to speak directly with a site foreman to secure access to a group of builders. The LR explained: *“Sometimes I had to ring four times or just turn up in person before anyone would listen. It could feel pushy, but once they heard what I had to say, it worked.”*

**Contributions:** Across the project, the LR engaged with 14 community groups and organisations through 39 visits, involving 276 individuals across diverse geographic locations, ages, ethnicities, literacy levels, and socio-economic backgrounds. She also facilitated over 700 social media interactions and recruited two members of the PRG and three members of the CAG.

**Challenges and lessons learned:** The dedicated LR role enabled broader and deeper PPIE activity than would otherwise have been achievable. She was given freedom to innovate and work creatively, however, appropriate boundaries and line management were required. For example, careful consideration was required regarding what could be shared on social media, given the risk of contamination in a clinical trial context.

Engaging individuals who were not typically involved in research was time-intensive, but the dedicated LR made this possible and yielded substantial benefits. Building trust sometimes meant spending time outside the scope of the project. As the Lay Researcher reflected: *“Building trust sometimes meant letting the group lead, even if that meant spending an hour on toothache stories before we got anywhere near the project.”*

Managing expectations of community members was a challenge. Some community members assumed the project could offer dental advice, free treatment, or resources like toothbrushes. The LR recalled: *“It was hard having to say no without losing their trust. I had to explain our remit again and again.”*

### Layer 2: patient reference group

The PRG was established early in RETURN. Two members had contributed previously to oral health- research and were recruited via existing relationships; two further members were recruited by the LR through GE. Recruitment was purposive, based on members’ ability to engage with PRG tasks and represent perspectives relevant to the RETURN population. PRG meetings were chaired by the LR, with research staff in attendance. Agendas were co-developed. The PRG comprised three women and one man, aged 60–70 years-old. Three identified as White British and one as British Indian. The PRG reflected a hybrid form of ‘professionalised PPIE’: some members brought research literacy, others community insight. Although not socio-economically representative of the target RETURN population, their combined expertise enabled them to advocate, anticipate and interpret community perspectives effectively.

The PRG met 10 times at key programme milestones. Terms of reference were co-developed (Additional File 1), and meetings were audio-recorded and minuted. Activities included: reviewing patient-facing materials; co-developing the intervention; contributing to monthly recruitment newsletters; refining questionnaire wording; shaping recruitment strategies; and advising on dissemination.

**Fig. 2:**
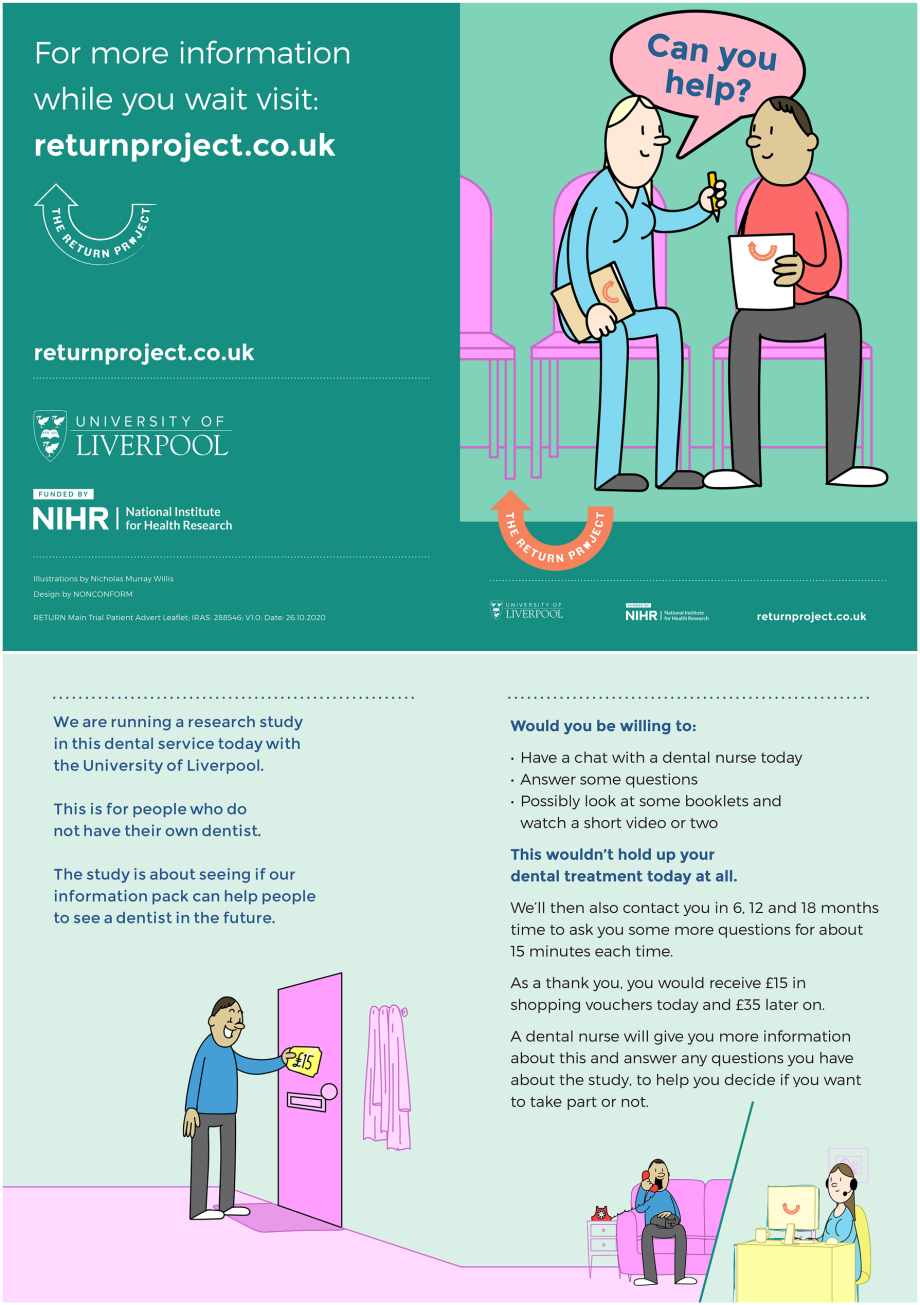
Approach leaflet co-produced with the PRG

**Contributions:** PRG input added value in several ways. For example, while all PPIE layers felt the Patient Information Sheet and Consent form (PISC) was overly long and complex (Additional File 2), the PRG recognised that much of the wording was non-negotiable due to Clinical Trials Unit procedures and GDPR legislation. They therefore recommended a concise “approach” leaflet to summarise the trial before formal consent. As one member noted: “*We knew we couldn’t change all the wording because of research governance requirements, so the approach leaflet made the trial accessible within the rules we had to follow*” (See Fig. 2).

A further example of their contribution concerned a research governance issue arising during negotiations with an external organisation holding trial outcome data. Mid-trial, the organisation proposed regulatory changes that would have required re-consenting trial participants, with significant implications for burden and feasibility. Drawing on their familiarity with research processes and their role as patient representatives, the PRG were invited into discussions. They proposed a proportionate alternative that met governance requirements while keeping participants’ interests central - updating trial information via the study website. Their involvement ensured the patient perspective was embedded in decision-making and appropriately weighted in negotiations. The RETURN Programme Manager reflected:

*Having the PRG involved in the governance problems we encountered when trying to make this complex trial work was vital. They were able to present pragmatic solutions from the patients’ perspective and remind stakeholders of the importance of proportionality and feasibility. What’s more, they were really listened to. It felt like a real David and Goliath moment.*

**Challenges and lessons learned: **One strength of the PRG was their awareness of trial constraints resulting in suggestions that were highly pragmatic and straightforward to implement. However, this had potential to limit more radical or unconventional ideas that were brought about through other PPIE layers.

PRG advice sometimes contrasted with feedback from other layers; for example, while the PRG suggested the PISC was long, complex but acceptable, feedback from the CAG and GE was that it was incomprehensible and not fit for purpose.

### Layer 3: community advisory group

A key purpose of the CAG was to embed lived experience in intervention co-development so materials reflected the social identity of urgent care users. Recruitment strategies included local newspaper adverts; local radio; Facebook; posters in public spaces; and GE (Layer 4). This generated a broadly representative group, including individuals receiving State Benefits, on low incomes, and living in areas of high deprivation. All those who expressed an interest in becoming a CAG member were invited to join; there was no competitive selection or cap on membership. Inclusion was based solely on experience of attending (or not attending) dental services.

The CAG comprised nine members (three male, six female), aged 28 to 60. Most identified as White British, with one Black British member. None had prior research involvement experience. The group met bi-monthly for two-years, primarily for intervention co-development. Relationships developed over time and their input continued on an ad hoc basis at later stages of the programme. Terms of reference were co-drafted (Additional File 3) and sessions were recorded but not minuted.

To maximise contribution, the research team prioritised relationship-building, used varied participatory approaches, and adapted methods to group needs. The longitudinal design allowed time for reflection and confidence-building, enabling deeper insights. For example, one member waited until the final session to share photographs and a personal story about dental visits, illustrating the importance of trust and support.

Over time the CAG developed a peer-support function alongside co-production, as illustrated in the exchange below:

*CAG member 1: Our kids can’t believe I do this [attend CAG meetings], knowing I’m terrified and my kids have got lovely teeth, “Mum you’re terrified” and I’m going “I don’t care, I’m going.*

*CAG member 2: Its turning fears into a positivity*

*CAG member 3: …. I’ve got this big fear, but I feel comforted by coming to this, and hopefully it’ll help other people and us because that’s another good feeling I’ve got about it.*

This dynamic not only benefited members, but provided rich contextual insight into barriers to dental attendance unlikely to emerge through short-term or conventional PPIE approaches. The CAG also reviewed participant-facing material.

**Contributions:** CAG members contributed stories and photographs directly to the RETURN intervention, with some appearing in intervention videos (Additional File 4). Rather than responding to pre-prepared drafts, they shaped the intervention’s foundations, ensuring it was rooted in lived experience.

**Challenges and lessons learned:** Working without set agendas encouraged open discussion but required skilled facilitation to manage dynamics and ensure inclusion. Some members initially lacked confidence, questioning whether their views were ‘helpful’: “*I don’t know if what I want to say is going to help anyone, as it’s just what I think, and it might be a bit silly*”, requiring reassurance from facilitators and peers. On one occasion, a member was not well received by others, requiring sensitive management by the team.

Recruitment and engagement was labour-intensive, involving multiple strategies and one-to-one conversations before the first meeting to build rapport and trust. While demanding, this investment was essential to create a safe, productive space where people without prior research experience could engage meaningfully.

### Layer 4: grassroots engagement

GE aimed to capture authentic perspectives through inclusive engagement in naturalistic community settings. While the PRG and CAG offered structured, ongoing involvement, GE was designed to reach people unlikely to join formal groups but whose voices were crucial to inclusive representation. It relied heavily on the LR proactively building community connections, often using snowballing via local leaders to expand networks. Many contributors had no prior research experience.

**Fig. 3:**
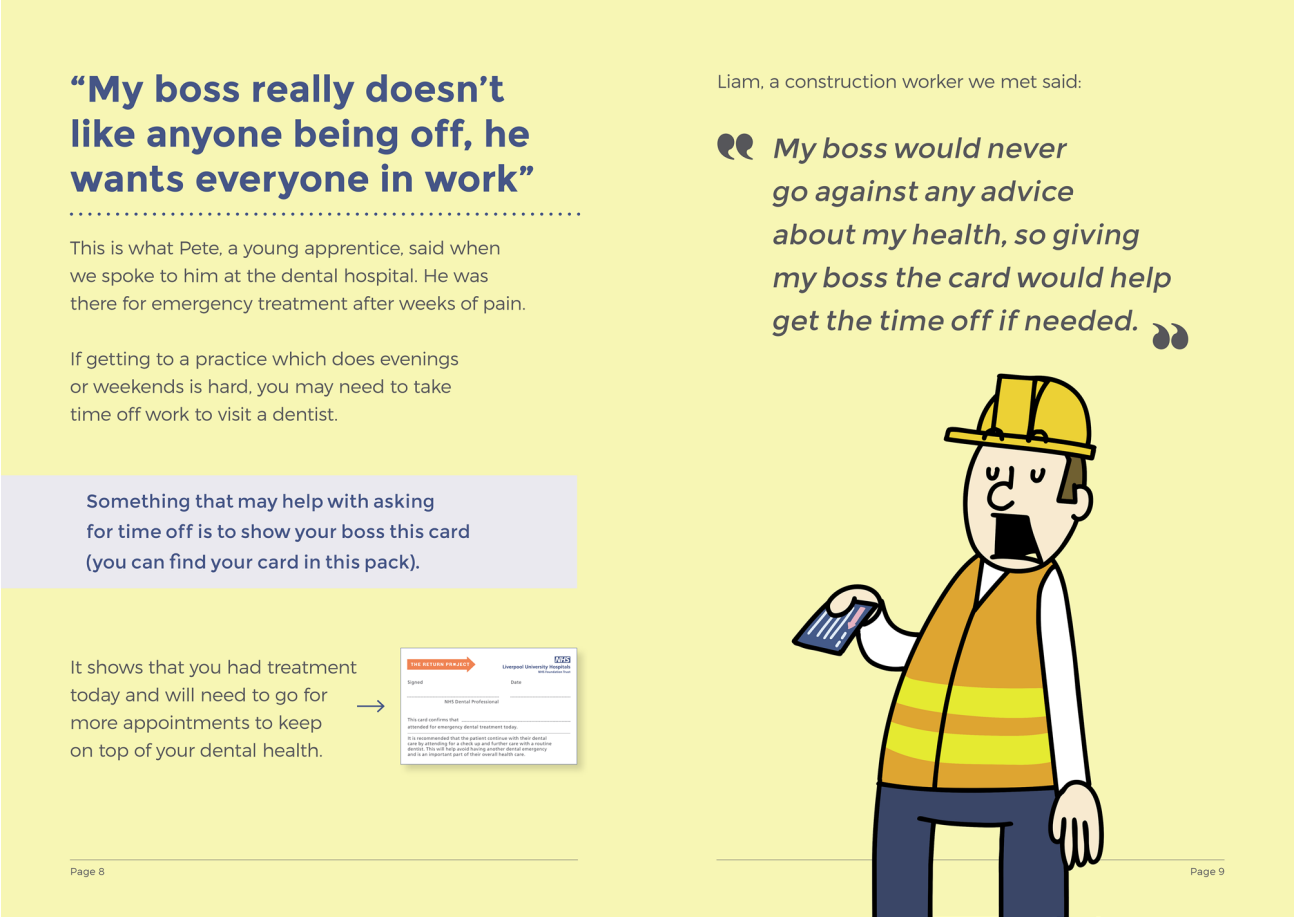
Excerpt from ‘time’ RETURN intervention, influenced by a GE visit to a construction site

Outreach and intervention development were iterative and responsive to emerging findings. For example, male construction workers (identified as prominent urgent care users in the ethnographic workstream) were engaged through informal lunchtime conversations on their building site. These insights directly informed the “Time” booklet within the RETURN intervention (Fig. 3).

Engagement methods were deliberately adaptive across diverse contexts (Table 1). No two settings were the same, requiring continuous adaptation of style, tone, and activity

**Table 1:** Summary of grassroots engagement RETURN PPIE activities

Organisation	Reason for approach	Type of activity	Number of visits	Description of PPIE partners	Mode of data collection	Pathway to access
Community Setting 1.	Anticipated a mix of local people, with high representation of those from low socio-economic status (SES) backgrounds	Short, informal conversations about dental visiting in an allocated room on site. Opportunistically approached people attending the setting	5	12 adults, mostly unemployed	Field notes written after visits	LR engagement
Community Setting 2.	Opportunity to engage with older people	Online meeting, informal feedback on intervention materials	1	15 adults over 50	Field notes written after visits	LR engagement
Community Setting 3.	Prolific local sporting charity with a focus on inequalities and health, anticipated opportunity to speak to men	Informal conversations while playing football; group discussions with military veterans about intervention prototypes; recruitment of a community lead for RETURN videos; newsletter updates; website development input	2 plus frequent email contact	16 adults − 15 men, 1 woman	Field notes written after visits	LR advised on organisation though a different community organisation, email and phone contact to identify the right organisational contact; emphasised inequalities focus to align with charity’s priorities. Given permission to engage with various groups under the organisation’s umbrella
Community Setting 4.	Anticipated opportunity to engage men from low SES backgrounds and those not attending routine dental care	One off visits for informal conversations about dental visiting plus feedback on intervention prototypes during lunch break in canteen	1	10 male adults, aged 18–37	Field notes written after visits	LR researched organisation, initial in-person approach to foreman, followed by permission and email arrangement
Community Setting 5.	To engage younger adults (low SES) not in full-time education or employment	Pre-designed structured workshops using quizzes, journey mapping, photo elicitation, Q&A sessions, design tasks, and feedback on prototypes/materials. Sessions were delivered as part of their course, and pre-agreed with course leaders; newsletter updates; website development input	11 plus frequent email contact	94 young adults aged 18–25	Photographs, drawings, written feedback and field notes written after visits	LR advised of organisation through a PPIE contact, emails to session coordinator (after some time investigating the best person to speak to) followed by in-person meeting; permission to attend gained, and agreed reciprocal arrangement for dentist to attend sessions to answer general questions
Community Setting 6.	Locally respected community group, anticipated opportunity to reach women from low SES backgrounds	Informal opportunistic group discussions over lunch at Ladies Friendship Group; group feedback on trial questionnaire; recruited a member of the PRG, newsletter updates	2 plus frequent email contact	20 adult women aged 40–70	Field notes written after visits	LR identified organisation via social media; phone contact until group leader reached and several phone calls until permission gained to attend
Community Setting 7.	Anticipated opportunity to connect with older women	Informal opportunistic conversations about general dental attendance, people approached opportunistically; recruited a member of the PRG; newsletter updates	2	8 adult women aged 55+	Field notes written after visits	LR informed of organization through a community contact, phone contact with group lead and permission gained
Community Setting 8.	Prolific local sporting charity, anticipated opportunity to engage with younger men	Informal opportunistic conversations alongside the activities of the community group (a singer)	1	22 adults − 19 women, 3 men	Field notes written after visits	LR informed of organisation through a community contact, several phone calls, in-person meetings were required to gain permission
Community Setting 9.	Anticipated opportunity to engage with families from low SES backgrounds	One-off opportunistic informal conversations with members of the local community whilst they were attending an indoor fair, recruitment of CAG member	1	12 adults	Field notes written after visits	LR informed of organisation through a community contact, telephone and email contact until permission given to attend
Community Setting 10.	To engage with leaders of local community groups in order to explore options to expand PPIE network, and to improve cultural diversity within intervention materials	Attending pre-arranged structured community champion meetings, with a slot to explain RETURN; newsletter updates	1	7 community group leaders	Notes taken during meeting and field notes written after visit, and follow-up emails	LR informed of organization through a PRG member, contacts by email and in person meeting to explain the project.
Community Setting 11.	To engage with staff members of local publicly funded organisations	Online meeting where feedback was asked for on intervention materials that were sent in advance; online focus group approach; newsletter updates; face to face wellbeing event attendance (with a stall showcasing the intervention) to engage in opportunistic discussions about dissemination	3	36 members of staff	Field notes written after visits.	LR informed of forum through other community groups. Access achieved through emails and phone calls (during Covid −19 pandemic)
Community Setting 12.	Anticipated opportunity to engage with people from low SES backgrounds about dissemination of trial results	Opportunistic informal group discussed with service users attending the café; pre-arranged informal discussions with the leaders	6	4 staff members (all female) 21 service users − 15 women, 6 men	Field notes written after visits. With staff members, notes taken during meetings and then written up into field notes.	LR researched community groups in low SES areas, contact by email, followed by in-person meeting at the University with group leader.
Community Setting 13.	Anticipated opportunity to engage with adults from low SES backgrounds	One-off opportunistic informal conversations at a drop in-food bank	1	10 + adults - mixed ethnicity aged 18 to 70.	Field notes written after visit	RETURN researcher identified organisation after initial search of local community groups, telephone call to explain purpose and permission for visit.
Community Setting 14.	Anticipated opportunity to engage with adults from low SES backgrounds	One-of opportunistic informal conversations during coffee and toast break at a jeweley making session.	1	10 adults − 1 man and 9 women	Field notes written after visit	RETURN researcher identified organisation after initial search of local community groups, telephone call to explain purpose and permission for visit.

**Fig. 4:**
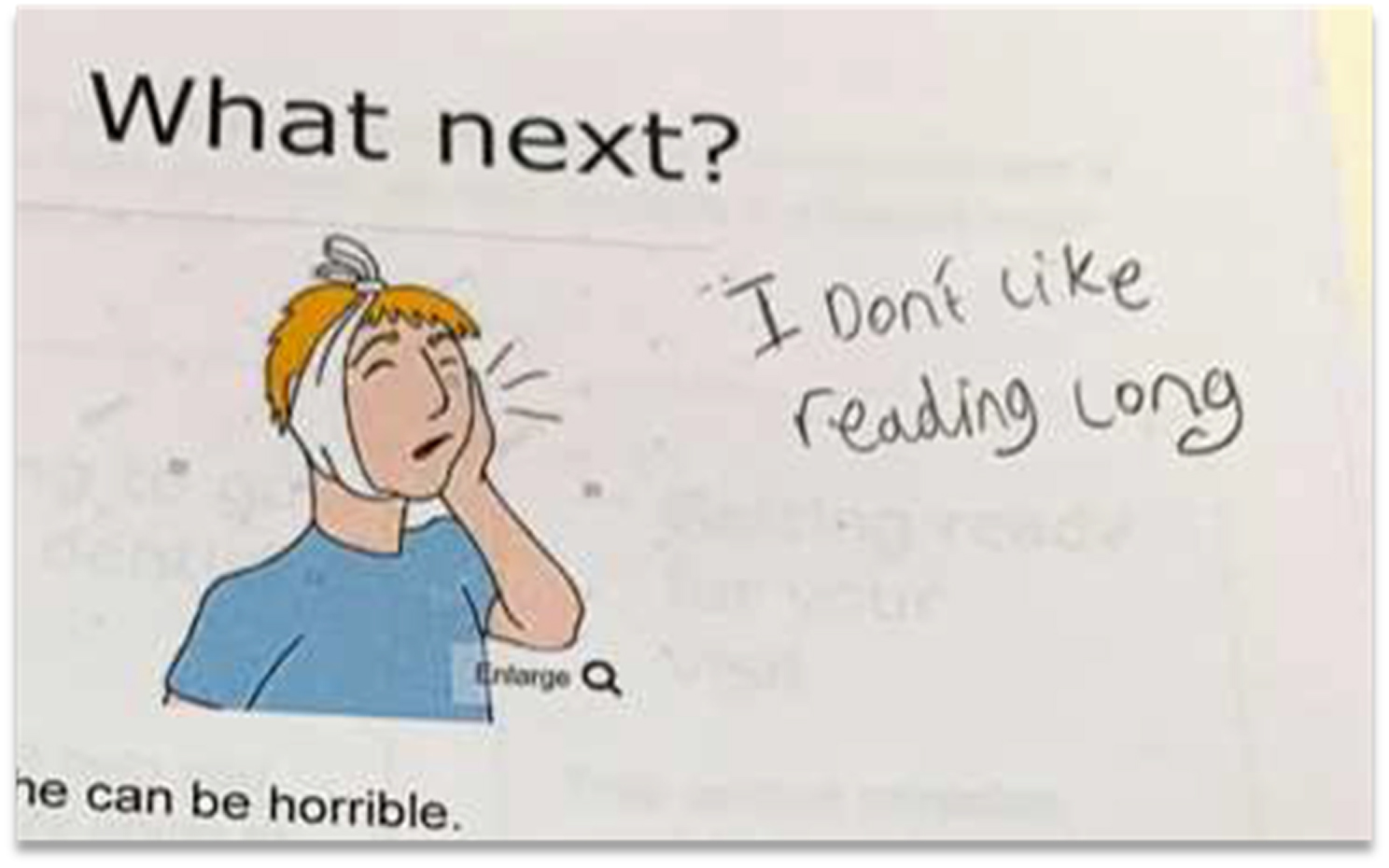
Image of feedback from grassroots engagement

GE generated rich, candid feedback. Early intervention iterations were returned with comments such as *“I don’t like reading long”* (Fig. 4) - a level of directness and honesty not seen in the other layers. The brevity and one-off nature of encounters mirrored the opportunistic way the intervention would be delivered. Feedback consistently emphasised practical solutions and ensured materials and processes resonated with real-life circumstances.

**Contributions:** GE made three distinctive contributions. First, it broadened inclusivity by engaging people who were unlikely to participate in structured forums. Second, it provided a “real-world sense-check” grounding intervention materials in lived realities. Third, its immediacy mirrored the opportunistic nature of RETURN intervention delivery; conversations were brief and pragmatic, generating a candour not seen in other PPIE layers.

**Challenges and lessons learned:** GE was resource-intensive and relied heavily on the initiative and persistence of the LR. Building trust in community settings required sensitivity and repeated contact; misjudged approaches risked closing opportunities for further engagement. Activities were informal and typically captured as conversational snapshots, shaped by who was present on the day. While spontaneity was a strength, documentation of encounters was necessarily light, relying on field notes, photographs, and drawings.

Coordinating GE across sites and tailoring approaches required sustained time and effort. Contributors were diverse, including individuals from marginalised backgrounds and some could be considered vulnerable. In one instance, a contributor became distressed during a drawing activity, highlighting ethical challenges in participatory work and the importance of community gatekeepers. Gatekeepers supported safe, sensitive engagement and facilitated trust in groups who might otherwise have not engaged.

Keys differences between the groups are summarised in Table 2.

**Table 2:** Overview of the PPIE structure and key differences between the layers

Layer	Purpose	Membership profile	Recruitment and participation	Frequency of Engagement	Communication Style	Typical Activities	Payment for involvement	Type of Input
Patient Reference Group	Provide strategic oversight, ensure patient perspective in trial governance, conduct and materials	Experienced PPIE contributors with prior research or community engagement	Open call via existing PPIE networks and through community group leader networks; participation based on contributor interest, confidence, and comfort with strategic tasks	Quarterly or as per the needs of the research	Online or in person ‘meeting-style’ engagement, formal agendas, PowerPoint presentations, structured minutes, documentation, follow-up actions	Reviewing trial documents, advising on communication strategy, providing advice on the intervention, identifying opportunities for PPIE input within the trial context	Yes, according to INVOLVE suggested, plus travel and refreshments	Strategic insight
Community Advisory Group	Co-develop intervention materials, ensure cultural and contextual relevance	Local residents with lived experience relevant to the trial population	Direct recruitment via social media, newspaper and radio adverts, community organisations and public avenues plus snowballing methods; participation based on lived experience and community knowledge	Bi-monthly during the developmental phase of the research. Ad hoc thereafter.	In-person, interactive workshops, focus groups, participatory methods (photovoice and photo elicitation), use of visual aids, iterative feedback loops, verbal summaries for accessibility	Co-development (including starring in videos, offering lived experience and sharing photographs), suggesting adaptations, identifying and contextualising barriers, providing guidance on tone, language and reach.	Yes, according to INVOLVE suggested rates, plus travel and refreshments	Lived experience & co-design
Grassroots Engagement	Reach seldom-heard voices, surface new perspectives	Individuals opportunistically encountered in community settings/community centered-social media	Opportunistic recruitment through established community group leaders (gatekeepers) plus social media; participation based on demographic representation of trial population	Ad hoc	Conversational, in-situ or online discussions, culturally sensitive and jargon-free, feedback captured immediately	Short, informal conversations, gathering quick feedback on materials or concepts	None. Refreshments provided, plus benefits such as non-clinical Q&A sessions with a dentist	Community insights

## Discussion

This paper presents a case study of how working with different people by simultaneously using complementary PPIE approaches supports authenticity and inclusivity. This approach contrasts with the often-binary portrayal of public engagement (PE - awareness-raising and informal dialogue) and patient and public involvement (PPI - structured formal partnerships to influence research) as separate entities [39]. More recently PPI and PE have been viewed as complementary fields that can be strategically aligned with research objectives [39]. The RETURN PPIE strategy provides an example of this in practice and demonstrates how combining different forms of involvement within the same project can provide both wide and deep engagement.

Previous work has highlighted the limitations of a “one size fits all” approach to PPIE [29, 40, 41], including its potential to de-democratise involvement. Greenhalgh et al.’s work further extends this critique, concluding that many PPIE models are aspirational, context-specific, and rarely transferable beyond their original setting [22]. They argue that effective PPIE requires flexible, modular approaches that are co-designed and adapted to local contexts. By explicitly addressing both the “*who*” and the “*how*” of involvement – which is often under-reported - this case study offers a practical illustration of a PPIE approach that supports flexibility and iteration, particularly where research objectives are inequalities focused.

In RETURN, this approach enabled the inclusion of established contributors, who brought experience of prior research involvement or community representation, alongside contributors with experiential knowledge of dental care and (non)attendance. Combining experiential knowledge with other skills and lived experience created opportunities for contributors to challenge assumptions and influence the research in ways unlikely to arise from a single form of involvement [42]. Importantly, it represented a purposeful widening of inclusion, guided by research objectives and the principle that those most affected by the research should have a meaningful voice in shaping it.

The value of our layered approach lay not in the volume of engagement, but in aligning different forms of contribution with contributors’ insights, capacities and preferences. This enabled socio-economic inclusion without requiring all contributors to engage in the same way. However, the model was resource-intensive, requiring sustained time and flexibility, particularly through the dedicated LR role and the long-term facilitation of multiple PPIE streams. Approximately £70,000 (around 3% of the overall RETURN grant, including funds for the LR role) was allocated to PPIE in RETURN plus additional supervision time. While this level of investment may not be possible for all studies, the underlying ethos - matching multiple forms of involvement to research aims and contributor expertise - can be applied proportionately where PPIE resources are available.

The investment enabled contributions unlikely to emerge from a single PPIE model and the layers operated concurrently rather than sequentially, avoiding the privileging of particular perspectives and allowing insights to challenge and contextualise one another. One notable outcome was successful recruitment and retention of RETURN trial participants living in areas of high deprivation, with over half residing in neighbourhoods ranked within the most deprived decile nationally (Index of Multiple Deprivation decile 1) [43]. Although recruitment and retention is multifactorial, this outcome is consistent with, and may have been supported by, our layered PPIE approach.

Reflecting on this experience, the research team would adopt a layered approach again, as concurrent PPIE layers widened opportunities for involvement. Future studies would benefit from making this rationale explicit from the outset and from clearer resourcing for coordination and cross-layer reflection to improve inequalities focused PPIE.

### Limitations of the approach

As a case study of a single, large, inequalities-focused programme of research, these findings are context-specific and not directly generalisable. While we describe perceived benefits of the layered PPIE approach, it is not possible to isolate the specific contribution of individual PPIE activities to trial outcomes. The approach was also closely tied to the skills, persistence, and contextual knowledge of a single LR, which may limit the transferability where comparable capacity is absent.

A key tension in RETURN arose as contributors, including the LR, became increasingly socialised into research norms. Although lay knowledge was intended to be valued on its own terms [33], the LR gradually positioned herself as an equal member of the research team, reflecting immersion in the academic environment. Similarly, the CAG chose to move meetings from community settings to the university, signally a shift towards more formalised engagement. As noted by Thompson et al. [44] professionalisation may lead contributors to support rather than challenge researchers, highlighting the difficulty of integrating lay expertise while preserving its independent, critical perspective, particularly in long-term projects.

Variation in intensity and format across layers also meant that documentation and synthesis of PPIE input were not uniform, potentially influencing how insights were prioritised and interpreted.

Recruitment methods may have introduced bias. For example, CAG members were recruited primarily through a public campaign inviting individuals to contact the research team. While open, this approach required a level of literacy, relied on people having access to a telephone (or credit to make a call) or the internet, and the confidence to approach a university research team, all of which may have shaped who ultimately volunteered. Nonetheless, none of our CAG members had prior research involvement experience, suggesting some success in widening participation. The GE workstream helped mitigate this limitation by taking engagement into community settings.

The PRG comprised older adults only, reflecting wider challenges in recruiting younger people into sustained, formal PPIE roles. Despite efforts to increase flexibility, longitudinal involvement may be less feasible for younger adults with competing commitments.

Finally, engagement with ethnically diverse communities progressed slowly. Structural barriers, alongside the positionality of the LR as White British, were recognised as potential constraints when building trust in some communities.

## Conclusion

The RETURN PPIE strategy demonstrates that layered approaches are an effective way to strengthen inclusivity in research. By integrating multiple forms of involvement, we created a structure that spanned the socio-economic spectrum, engaging both experienced representatives and harder-to-involve communities. This approach shows how a PPIE strategy can widen diversity while still maintaining depth and rigour.

## Electronic supplementary material

Below is the link to the electronic supplementary material.

Supplementary Material 1

Supplementary Material 2

Supplementary Material 3

Supplementary Material 4

## Data Availability

Due to ethical and confidentiality considerations, the full qualitative data set cannot be shared. However, summarized findings and selected anonymized excerpts may be made available upon reasonable request.

## Declarations

### Authors’ information

The authors of this paper include academic, lay, and patient contributors involved in the RETURN study. Dr Victoria Lowers is a mixed-methods researcher in dental public health and contributed to the design and ongoing support of the PPIE work within the RETURN study over approximately seven years. Margaret Stanley was employed as the lay researcher on the RETURN study and holds a Master’s degree in Oral History; she led the day-to-day delivery of the PPIE workstream throughout the study. Edwin Morgan, Jyoti Vithlani, and Ruth Horsley are patient representatives who contributed to the RETURN study through their involvement in the patient reference group. Professor Rebecca Harris is Professor of Dental Public Health and Chief Investigator for the RETURN study.

## Abbreviations

PPIE: Patient and public involvement and engagement
RETURN: Intervention to reduce inequalities in the uptake of routine dental care study
NIHR: National Institute for Health and Social Care Research
LR: Lay researcher
PRG: Patient reference group
CAG: Community advisory group
GE: Grassroots engagement
PE: Patient engagement
PPI: Patient and public involvement
