# Supplementary material for: Layering perspectives: a structured approach to meaningful patient and public involvement and engagement in the RETURN dental trial
Source: Res Involv Engagem. 2026 Mar 7;12:44. doi: 10.1186/s40900-026-00857-w (PMC13081612; doi:10.1186/s40900-026-00857-w)
Supplement: Supplementary file 1 — Supplementary Material 1 [file 40900_2026_857_MOESM1_ESM.pdf]

RETURN Patient Reference Group: Terms of Reference

1. INTRODUCTION

The purpose of this document is to describe the membership, roles, responsibilities, for the **Patient Reference Group (PRG)** for the RETURN programme. This includes the timing of meetings, frequency and format of meetings and relationships with other programme committees.

2. ROLES AND RESPONSIBILITIES

2.1 Aims of the PRG

The broad remit of the PRG is to provide advice from a Patient's / Public perspective and remain involved in the organisation and governance of the Return project.

2.2 Roles

- To provide in-depth insights from a Patient / Public perspective as the project progresses.
- Examine initial observations to secure clarification on emerging themes to provide insight and direction for field work and wider Public Engagement activities.
- Work with the Ethnographer to ensure that findings and reports portray an accurate representation of the views of participants.
- Give feedback on and guide the format of intervention materials
- Provide ongoing support with the scrutiny of all patient facing materials to ensure maximum impact and acceptability.
- Provide advice and support about dissemination of findings
- Inform and support the recruitment of members of the Community Action Group (CAG)

2.3 Responsibilities

- At least one member of the PRG to attend Project Management Group (PMG) meetings to give a Patient / Public perspective in discussions.
- To provide advice through discussion at meetings or outside meetings via e mail or telephone
- To support the entire project (5 years 9 months) and oversee its development
- All members will respect full confidentiality and ensure that the project maintains control over all materials and release of information concerning the research.
- We ask the PRG to remember that we are bound by *Chatham House rules.* This means that *participants may not discuss any identifying details of other members outside of the group*. This aims to ensure that members have the opportunity to air their views within meetings without concerns that their identity or personal views will be discussed outside of the group.

3.0 MEMBERSHIP

- 4 Independent members have been recruited to form a committee with a wide range of skills.
- Members will be supported with the provision of training where available.

- Any materials which will provide additional support will be sent to members by Patient Public Representative (Margaret Stanley) who will act as lead for this committee.
- Members will be paid £75 for their participation in the meeting which may include the reading of materials sent in advance for comment.

RETURN Patient Reference Group: Terms of Reference (2)

4. ORGANISATION OF PRG MEETINGS

4.1 Frequency of Meetings

The PRG will meet up to four times per year,

- Initially the responsibility for organising and chairing meetings rests with the Chief Investigator (Rebecca Harris). This will later transfer to the Patient Public Representative (Margaret Stanley).
- Meetings will be arranged in line with the key checkpoints within the project to inform the key decision-making process.

4.2 Attendance at meetings

- All members of the PRG will be invited. A series of dates will be offered to afford as many members as possible the opportunity to attend.

4.2 Format of Meetings

- Meetings will be face to face or online and organisation of meetings will be supported by the RETURN Programme Administrator (Helen Rowe).
- N.B. In light of developments due to the Covid-19 pandemic, meetings will take place according to guidelines regarding University of Liverpool safe working practices. Meetings will take place using online remote access as required.

- The PRG agenda and relevant supporting documents, including minutes of the previous meeting, will be circulated before the meeting to allow time for the review.

- PRG members who will not be able to attend the meeting should send apologies.

4.3 Minutes of the PSC Meeting

- The RETURN Programme Administrator (Helen Rowe) will be responsible for taking minutes of the meetings. Minutes will subsequently be circulated to all members of the group.
