# Supplementary material for: Layering perspectives: a structured approach to meaningful patient and public involvement and engagement in the RETURN dental trial
Source: Res Involv Engagem. 2026 Mar 7;12:44. doi: 10.1186/s40900-026-00857-w (PMC13081612; doi:10.1186/s40900-026-00857-w)
Supplement: Supplementary file 2 — Supplementary Material 2 [file 40900_2026_857_MOESM2_ESM.pdf]

Information for patients about the RETURN study

What is the RETURN study?

Some people put off going to a dentist until they have a problem. This study is about helping people to make regular dental visits.

The National Institute for Health Research (NIHR) have given money to a research team to develop a pack of information which could be given out to people who visit the dentist when they have a problem.

- We are testing the pack in a clinical trial to see if it helps people who only visit the dentist when they have a problem.
- We are looking for 1,180 people to take part who live in Cheshire and Merseyside and are aged 18 years or over and use emergency dental services
- Half of the people taking part in the study will be given the RETURN pack as well as the normal care given at their emergency dental appointment – and half will not get the RETURN pack but only care as normal.
- If we find it helps people, the pack would be used in other areas of the UK.

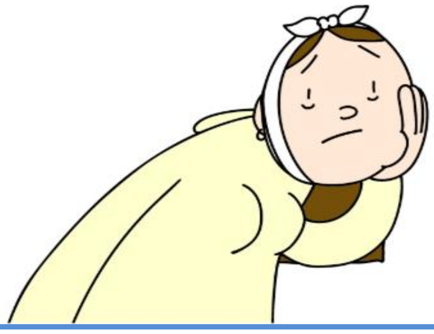

Taking part will not delay your care today.

Do I have to take part?

- No. Taking part is voluntary and you can also choose to stop at any time without saying why.
- If you don't want to take part, you will receive your dental care as normal today. This would not affect any further treatment you receive.

What would I have to do?

- If you decide to take part you will be asked to sign a consent form to confirm this.
- We will then ask you some questions to double check that you are suitable for the study.
- You will then be asked to answer some questions about you and your dental care. This takes about 15 minutes.
- It will then be decided if you will be in the group who receive the information pack.

This is decided by chance, by a computer

If you are given the pack, you will answer some questions again for about 5 minutes. Someone will talk you through the pack and show you one or two 2-3 minute videos. This all takes about 15 minutes. This group also get a text a couple of weeks later with an online link to the pack.

Both groups will answer 5 more mins of questions today and watch a 2-minute video which says 'Thank you' and lets you know when we will get in touch again.

- **After 6, 12 and 18 months from now everyone** will get a phone call to ask if you have gone to a dentist again, and about any treatment you have had. We will text first to arrange a good time for you. We will audio record the call. This will take about 10-15 mins.

What is in the RETURN pack?

We have worked with local people to develop the pack.

o There are several small booklets about reasons why people only visit the dentist when they have a problem, such as cost or anxiety (called 'barriers').

o There are also short videos with people talking about how they feel about that barrier. They also have an animation.

o There is a section to help you make plans to overcome the barriers and make an appointment.

o There is a lot of information such as about how to get help with the cost of treatment, and tips such as how to relax at the dentist.

o There are some pull out materials e.g. a card to show your employer that you need some time off work for an appointment, and information which you can show the dentist you visit, if you are anxious about dental care.

For 50 people taking part in the study the follow-up interview at 6, 12, and 18 months will be longer. What does this involve?

o For 35 people who get the pack and 15 who do not, the interviews at 6, 12 and 18 months will be longer so we can really listen to your story about visiting the dentist after today.

o Interviews will be up to 60 mins. If you would like to be interviewed in your own home or at another place, we can arrange this. Or we can do this by phone.

o We will audio record this interview. We will anonymise and store this data in the same way as other data collected. For more information visit www.returnproject.co.uk).

o Only some people will be invited to have a longer interview. You can opt out of this part if you want, and still take part in the rest.

The RETURN pack may be given to you before or after your dental care – it will not delay you care!

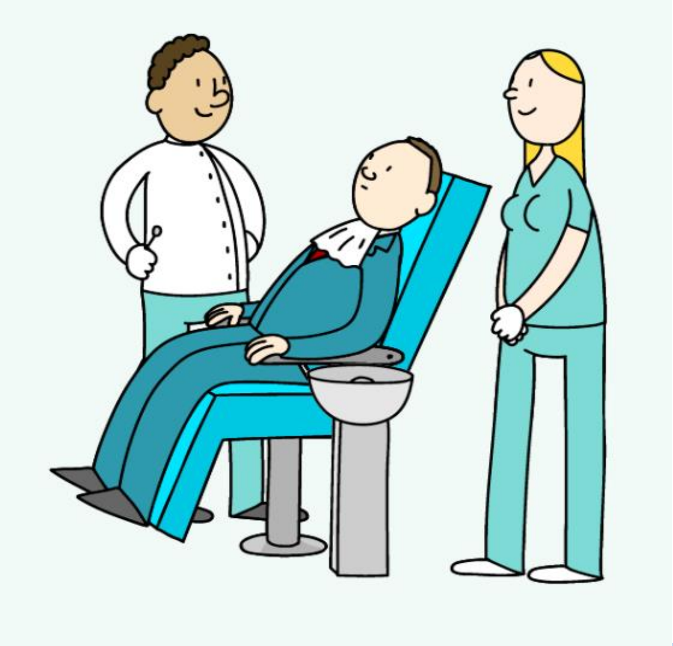

What expenses will I get?

Everyone will be given shopping vouchers for taking part in this study – even if they did not receive the pack.

The vouchers will be given when you complete these stages:

Timepoint	Voucher
Baseline (today)	£15
6-month follow-up	£10
12-month follow-up	£15
18-month follow-up	£10

You will be given the baseline voucher today and we will post the others to your home address.

Everyone taking part will also be entered into a prize draw to win a Tablet PC.

The winner will be drawn after all the 12-month follow-up calls are completed, which may be about 18 months from today.

- If you take part in a longer interview you will receive an extra £15 in shopping vouchers, each time you are interviewed.

What are the benefits and risks of taking part?

Taking part means that we will be able to know whether the pack is useful to use in the NHS for people who only visit the dentist when they have a problem.

We think that the RETURN pack may help people use dental services more regularly.

- Visiting a dentist early means the dentist can pick up problems early and mend things e.g. with a smaller filling rather than possibly losing the tooth.

We are not aware of any draw backs of receiving the RETURN information pack.

- People who are in the group who do not receive the pack will just receive their dental care and information in the usual way.

For people who didn't get the pack there are no more risks than not being in the study.

All dental teams you see will follow national guidance on preventing COVID-19 transmission e.g. use of Personal Protective Equipment etc.

What happens if I change my mind about taking part?

o If at any point you decide to stop taking part you will still get your usual care by the dental team. This will not affect the care you may receive in the future. You do not need to provide a reason for stopping being involved

o If you decide to stop taking part, we will use any information collected up until that time.

o If you don't wish to take part at any point after today, please contact a member of the team using the details on the top of page 1. Although we will not contact you again, we will check if you are happy for us to still collect your NHSBSA data. You could say 'No, thank you' to this bit as well if you wanted.

Who is running the study?

The University of Liverpool is the Sponsor of this study and is responsible for managing it. They are based in the UK. They have asked for the day-to-day running of the study to be carried out by teams based at the Liverpool Clinical Trials Centre (LCTC, part of the University of Liverpool) and Department of Public Health, Policy and Systems (PHPS, part of the University of Liverpool).

As an ethically approved study sponsored by the University of Liverpool, the RETURN study has full clinical trial insurance cover.

This study is funded by the National Institute for Health Research Programme Grants for Applied Research (ref: RP-PG-0616-20004).

FUNDED BY NIHR | National Institute for Health Research

Your dentist or dental team will not receive any personal payments for including you in this study.

The study has been reviewed by the Health Research Authority and the National Research Ethics Service Committee to make sure that the study is scientifically and ethically acceptable.

Abbreviations used in the next sections:
LCTC: Liverpool Clinical Trials Centre. Staff based at the University of Liverpool who run clinical studies.

NHSBSA: NHS Business Authority Service. The NHSBSA processes payment for NHS dental practices and analyses data including records of treatment you have received. If you agree to take part in the study they will use your name, date of birth and postcode to provide us with details of your dental visits and treatment.

PHPS: Staff based in the Department of Public Health, Policy and Systems at the University of Liverpool

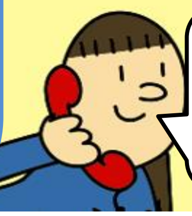

We will double-check if you still wish to take part in the study when we contact you 6-, 12- and 18-months from today's visit!

How will my information be collected and handled?

The University of Liverpool and the University of Leeds are the Data Controllers for this study and will need to use information from you and your dental records for this research project. This information may include:

- Name
- Date of birth
- Telephone number
- Email address
- Gender
- Information on your dental health, use of dental care and treatments you may have had.
- Age
- Level of education
- Employment status
- Benefit status
- Ethnicity
- Postal address

Individuals from the University of Liverpool (Study Sponsor), the LCTC and PHPS may look at your medical and research records to check the accuracy of the research study.

People who do not need to know who you are will not be able to see your name or contact details. Your data will have a code number instead. We will keep all information about you safe and secure.

The only time we might tell anyone what you have told us is if you were to give us information that worried us about you being at risk of harming yourself or other people. We would then follow safeguarding processes and might ask for contact details of your GP to contact them

Once we have finished the study, we will keep some of the data so we can check the results. We will write our reports in a way that no-one can work out that you took part in this study.

Records held at the Dental Hospital are not available through the NHSBSA.

If you are a patient at the Dental Hospital your name, date of birth and unique hospital identifier (RQ6) will be collected so that one of the PHPS researchers can collect routine records on dental services you have used since you visited today.

What are my choices about how my information is used?

We need to manage your records so the research is reliable. This means that we won't be able to let you see or change the data we hold about you.

The dental team will make sure that relevant information about the study is recorded for your care and to manage the quality of the study. They will also pass these details to the LCTC along with the information collected from you and your dental records.

Staff from the RETURN Sponsor (at the University of Liverpool), LCTC and PHPS may look at your dental and research records to check the information we have is correct.

The only people at the LCTC who will have access to information that identifies you will be people who need to review consent and the data collection process. Your name and contact details will be also given to the RETURN project team of researchers in PHPS, via a secure database held by the LCTC, so they can contact you for follow-up.

The LCTC and University of Leeds researchers who are analysing costs of care and any benefits of the pack (health economics) will look at nationally held data records on any dental practice services you have used during the study. This is done through the NHS Business Authority Service (NHSBSA). To do this, the LCTC will securely provide your name, gender, postcode and date of birth to the NHSBSA. This information provided to NHSBSA is known as a special category of information so this information will always be transferred securely.

You can stop being part of the study at any time, without giving a reason, but we will keep information about you that we already have.

If you choose to stop taking part in the study, we would like to continue collecting information about your health from the NHSBSA. If you do not want this to happen, tell us and we will stop. You can remain updated with how your data is used by going to www.returnproject.co.uk.

Information sharing for other research

When you agree to take part in a research study, the information about your health and care may be beneficial to researchers running other research studies in this organisation and in other organisations. These organisations may be universities, NHS organisations or companies involved in health and care research in this country or abroad. Your information will only be used by organisations and researchers to conduct research in accordance with the UK Policy Framework for Health and Social Care Research, or equivalent standards.

If you agree to take part in this study, you will have the option to take part in future research using your data saved from this study. Your data from this project will be anonymised (so that no-one can identify that it relates to you) before being provided to other researchers to use for a different study in the future. This is so the NHS can make the best use of money which is invested in research.

However, we will not use any data which might be linked to you personally in any future study, without getting in touch with you again to ask for your permission first.

You can also opt out of receiving any further contact from us about future studies if you would like to (for more information, visit www.returnproject.co.uk).

What happens at the end of the study?

The results will be presented at conferences and published in scientific journals so that we can explain to people working in dentistry, other researchers, policy makers and the public what our results have shown.

We will also publish the results on the website www.returnproject.co.uk

You will never be publicly identified as taking part in this study. This, and your data such as responses to questions will be confidential.

What if there is a problem?

If you have a concern about anything to do with this study, please speak to the dental team member named in the blue shaded box right at the top of this sheet who will do their best help you.

If you are still unhappy and wish to complain, you can do this by contacting:

Dental Practice Participants	Dental Hospital Participants
NHS England	Patient Advice and Complaints Team
0300 311 22 33	0151 706 4903
england.contactus@nhs.net	PALS@liverpoolft.nhs.uk

Where can I find out more about how my information is used?

Find out more from the RETURN website: www.returnproject.co.uk; at: http://www.hra.nhs.uk/information-about-patients; in the Health Research Authority leaflet available from: www.hra.nhs.uk/patientdataandresearch

Or you may contact either the University of Liverpool Data Protection Officer: LegalServices@liverpool.ac.uk or the University of Leeds Data Protection Officer: a.c.temple@leeds.ac.uk

Additional information

Every care will be taken in the course if this study. However, in the unlikely event that you are harmed by taking part in this research project (sponsored by the University of Liverpool), compensation may be available, and you may have to pay your related legal costs. Your dental care provider has a duty of care to you whether or not you agree to participate in this study and the study Sponsor accepts no liability for negligence on the part of your dental care provider’s employees. However, if you are harmed and this is due to someone’s negligence at your dental care provider, then you may have grounds for a legal action for compensation against the dental care provider where you are being treated, but you may have to pay for your legal costs.

FOR SITE USE ONLY:

Site Name:	
Participant Study Number	
Participant Initials:	Participant DOB: / /

Consent Form

To be completed by the participant:

Once you have read and understood each statement please write your initials in each box	Initial
Example: I have read and understood the information sheet for this study.	JS
1. I have read and understood the information sheet for this study. I have been able to ask questions. I have had these questions answered and I am happy with the answered I have been given.	
2. I understand that taking part is voluntary and that I am free to withdraw from the study at any time, without giving a reason, and without my care or legal rights being affected.	
3. I give my consent for a copy of this fully filled in consent form to be sent to the LCTC (where it will be kept in a secure location) to show that my consent was given.	
4. I understand that relevant sections of my dental records and any information collected during the study may be looked at by authorised individuals from the central study team, representatives of the Sponsor, and those listed in "How will my information be collected and handled?". I give permission for these individuals to have access to my records and data.	
5. I agree for the relevant data on my NHS dental treatments to be collected from the NHS Business Services Authority or where relevant directly from the Dental Hospital for the study period.	
6. I understand that my data (including any telephone transcripts, audio recordings and questionnaires) will be kept by the University of Liverpool, the University of Leeds, and at my dental practice / hospital in a confidential way for 10 years from the end of the study.	
7. I understand that I may be audio-recorded or observed when being given the pack and I give my consent to do this.	
8. I agree that the contact details I have given will be used by the research team to contact me to ask for follow up information and that any phone calls may be audio recorded (the caller will check with me first if I am happy with this).	
9. I agree to take part in the above study.	

The statements below are optional (you can still take part in the study even if you do not wish to agree to these):

10. I agree that I may be contacted to ask to be take part in a longer follow up interview after 6, 12 and 18 months which would be audio-recorded.
(if you agree to this statement provide your details below):

Telephone number:	
Email address:	

11. I understand that quotations from a transcript from an audiotape may be used as a quote in a publication, although I will not be able to be identified as giving the quote.

12. I agree that I may be contacted in the future about this or other related studies.
(if you agree to this statement provide your details below):

Telephone number:	
Email address:	

13. I agree to allow information or results arising from this study to be used in future healthcare and / or medical research providing my confidentiality is kept.

FOR SITE USE ONLY:

Site Name:	
Participant Study Number	
Participant Initials:	Participant DOB: / /

Consent Form

To be completed by the participant:

Participant full name (please print):			
Participant signature:		Date:	

To be completed by the Researcher (after participant has completed the form):

Researcher full name (please print):			
Researcher signature:		Date:	

Thank you for taking the time to read all the information about the study.

Should you decide to take part in the study you will be given a copy of the information sheet and a signed consent form to keep

Please file the original wet-ink copy in the RETURN Investigator Site File, and make three copies: one for the participant, one for the medical notes and one to be sent to the LCTC.

FUNDED BY NIHR | National Institute for Health Research
