# Supplementary material for: Layering perspectives: a structured approach to meaningful patient and public involvement and engagement in the RETURN dental trial
Source: Res Involv Engagem. 2026 Mar 7;12:44. doi: 10.1186/s40900-026-00857-w (PMC13081612; doi:10.1186/s40900-026-00857-w)
Supplement: Supplementary file 3 — Supplementary Material 3 [file 40900_2026_857_MOESM3_ESM.pdf]

What will a CAG meeting look like?

Each meeting will be about current stages of the project. Meetings will be facilitated by one or more researchers. Rather than taking minutes, these informal sessions will be audio recorded for use within the project. These recordings will be stored securely and confidentially.
CAG meetings are designed to be relaxed and informal with some basic points for discussion set by the RETURN team.
There is not a 'Chair' role within meetings, instead one of the researchers will facilitate discussion around the topics and materials of the meeting.
This means that all contributions are valid and all members should feel able to participate and have a fair share of discussion time.
The sessions are to be reflective where CAG meetings inform our research by listening to opinions from local people rather than searching for answers.
All participants in the meeting are expected to ensure that every CAG member can be heard in a non-judgmental and tolerant manner.

Contact details :
Please contact Helen Rowe the Return Project Administrator – for any admin queries e.g. expenses, apologies etc.
on 0151 794 8928 hrowe@liverpool.ac.uk

If you have any other questions please feel free to contact
Margaret Stanley 0151 795 5336 m.c.stanley@liverpool.ac.uk
Or Victoria Lowers 0151795 5316 lowers@liverpool.ac.uk

Community Advisory Group Terms of Reference

Describing the aims, guidelines, roles, responsibilities and meeting format for the Community Advisory Group (CAG) for the RETURN project.

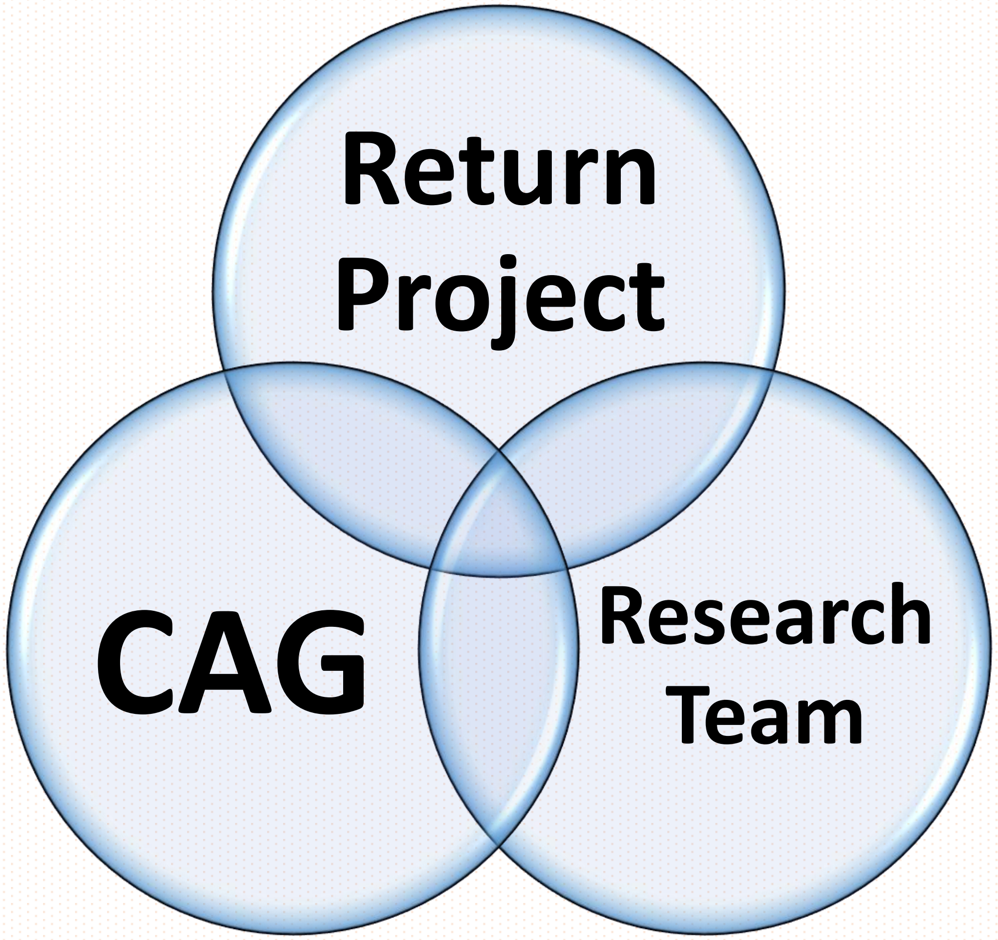

UNIVERSITY OF LIVERPOOL

FUNDED BY NIHR | National Institute for Health Research

Research

What do the research team do?

We are gathering information on using dental services so we can improve dental care and design and test an intervention.

Ultimately we hope to improve access to dental care and make it easier for people to use services when they are not in pain.

What will CAG members do?

CAG members help to create and maintain an atmosphere within meetings which ensures that all Return staff and CAG members feel comfortable and are able to contribute.

We will ask the CAG to help ensure the project materials are relevant to its audience. The CAG also have a vital role in the ongoing development of community activities, by designing and contributing materials and personal stories. We ask the CAG to remember that we are bound by *Chatham House rules.*

This means that *participants may not discuss any identifying details of other members outside of the group*.

This aims to ensure that members have the opportunity to air their views within meetings without concerns that their identity or personal stories will be discussed outside of the group.

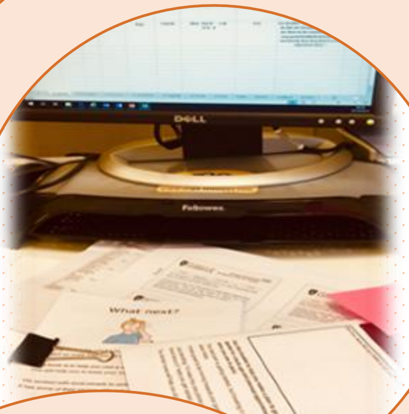

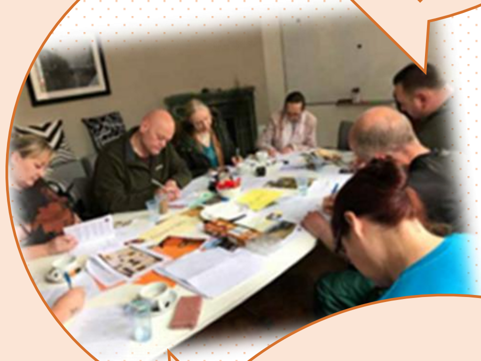

THE RETURN PROJECT

CAG

What are the main aims of the CAG ?

The CAG provides a public voice and community based opinions for the researchers. CAG members will contribute to and co-design, community-based activities.

We really value each individual member of the CAG because everyone will have their own story and point of view.

Building all of these experiences in to the material will help make the Return intervention relevant to local people.

The CAG will be asked to contribute to group discussions around the topic of dentistry, providing their own personal insights and sharing experiences . They could also help to recruit CAG members.

How will it work?

CAG members (who live/are involved in local communities in Liverpool/Merseyside) will be recruited using a variety of methods to create a group which is similar to the people living in Merseyside. No previous public engagement experience is needed.

CAG members receive payment for their time of £75 plus travel costs for meetings of up to 3 hours attended at the University or other local venue. This may also include time spent outside of meetings to give feedback on materials as the project progresses.

Meetings will usually be bi-monthly in support of key milestones within the project. Additional meetings may be scheduled (subject to CAG members' availability) as new developments arise.
